# Long-Term Exposure to Ozone and Life Expectancy in the United States, 2002 to 2008

**DOI:** 10.1097/MD.0000000000002474

**Published:** 2016-02-18

**Authors:** Chaoyang Li, Lina S. Balluz, Ambarish Vaidyanathan, Xiao-Jun Wen, Yongping Hao, Judith R. Qualters

**Affiliations:** From the Division of Environmental Hazards and Health Effects, National Center for Environmental Heath (CL, LSB, AV, JRQ); Division of Global HIV/AIDS, National Center for Global Heath (X-JW); and Division of Bacterial Disease, National Center for Immunization and Respiratory Diseases, Centers for Disease Control and Prevention, Atlanta, GA, USA (YH).

## Abstract

Long-term exposure to ground-level ozone is associated with increased risk of morbidity and mortality. The association remains uncertain between long-term exposure to ozone and life expectancy.

We assessed the associations between seasonal mean daily 8-hour maximum (8-hr max) ozone concentrations measured during the ozone monitoring seasons and life expectancy at birth in 3109 counties of the conterminous U.S. during 2002 to 2008. We used latent class growth analysis to identify latent classes of counties that had distinct mean levels and rates of change in ozone concentrations over the 7-year period and used linear regression analysis to determine differences in life expectancy by ozone levels.

We identified 3 classes of counties with distinct seasonal mean daily 8-hr max ozone concentrations and rates of change. When compared with the counties with the lowest ozone concentrations, the counties with the highest ozone concentrations had 1.7- and 1.4-year lower mean life expectancy in males and females (both *P* < 0.0001), respectively. The associations remained statistically significant after controlling for potential confounding effects of seasonal mean PM_2.5_ concentrations and other selected environmental, demographic, socio-economic, and health-related factors (both *P* < 0.0001). A 5 ppb higher ozone concentration was associated with 0.25 year lower life expectancy in males (95% CI: −0.30 to −0.19) and 0.21 year in females (95% CI: −0.25 to −0.17).

We identified 3 classes of counties with distinct mean levels and rates of change in ozone concentrations. Our findings suggest that long-term exposure to a higher ozone concentration may be associated with a lower life expectancy.

## INTRODUCTION

One of the most widespread air pollutants in the United States (U.S.) is ozone at the ground level.^1^ Studies have consistently shown that acute exposure to high ozone concentrations could harm the respiratory tract. Such harm could extend to declines in lung function, induction of inflammation, increases in respiratory symptoms and medication use in children with asthma, respiratory-related hospitalization, emergency room visits for chronic obstructive pulmonary disease and asthma, and an increase in incidence and hospital admissions for cardiovascular diseases.^2–5^ Growing evidence has shown that long-term exposure to ozone could also affect people's health.^6–8^

Life expectancy at birth is an important indicator of overall health status and quality of life and summarizes the mortality at all ages across the life span in a population. A reduction in exposure to ambient fine particulate matter (PM_2.5_) was associated with significant improvements in life expectancy during the 1980s and 1990s in the United States.^9^ But the effects of long-term exposure to ozone on life expectancy remain uncertain. Thus, our study had 2 objectives: first, we identified the clustering of county units according to the levels and rates of change in ozone concentrations between 2002 and 2008 in the conterminous U.S. Second, we sought to assess direct associations between long-term exposure to ground-level ozone and life expectancy.

## METHODS

We included 3109 conterminous U.S. counties in 48 states and the District of Columbia (excluding Alaska and Hawaii) in this study due to air pollution data availability. Data on modeled estimates of ozone and particulate matter concentrations, meteorological data, population data, and demographic characteristics at the county level are available from the National Environmental Public Health Tracking Network (Tracking Network) at the Centers for Disease Control and Prevention. The Tracking Network is a system of integrated health, exposure, and environmental hazard information and data obtained from a variety of national, state, and city sources.^10^ No ethical review was needed for this study because all data are aggregated at the county level without person-level identifiable information and publically accessible.

### Estimates of Ozone Concentrations and Other Environmental Factors

The Bayesian space-time downscaling (DS) fusion modeling approach, developed by the U.S. Environmental Protection Agency (EPA) and its partners,^11^ was used to generate predictions of ozone and PM_2.5_. The DS modeling approach combines gridded output from the Community Multi-scale Air Quality (CMAQ) model^12^ with monitoring measurements from Air Quality System, yielding more accurate and precise predictions of ozone and PM_2.5_ concentrations. The U.S. EPA used output from the CMAQ model executed at both 12 × 12 km and 36 × 36 km spatial resolutions to generate predictions for 2001 to 2006 for the eastern U.S. and used output from the CMAQ model executed at a 12 × 12 km spatial resolution to generate predictions for 2007 to 2008 for the entire conterminous U.S. Using the identical DS modeling approach and input, we generated daily mean 8-hour maximum (8-hr max) ozone concentrations (parts per billion or ppb) and daily 24-hour mean PM_2.5_ concentrations (micrograms per cubic meter or μg/m^3^) at the 2010 U.S. Census tract centroid locations over the entire conterminous U.S. for 2002 to 2008 in this study. Mean daily 8-hr max ozone concentrations were based on local ozone monitoring seasons, which vary by states. Seasonal mean estimates at county level were obtained using a population weighted approach, where tract populations were used to weigh daily tract level ozone and PM_2.5_ predictions.^13^ The county level estimates for ozone and PM_2.5_ concentrations in conterminous U.S. were validated carefully and are available at the Centers for Disease Control and Prevention Tracking Network.^10^ By using temperature data from the North American Land Data Assimilation System, we calculated the annual mean number of days with a heat index >90°F for the Tracking Network.^14^

### Life Expectancy Estimates in the U.S. Counties

We obtained life expectancy data from the University of Washington Institute of Health Metrics and Evaluation.^15^ Details of the methods and descriptive results on life expectancy estimates at the county-level have been published previously.^16,17^ In brief, life expectancy was estimated by a mixed effects Poisson regression with time, geospatial units, and demographic and socio-economic components using mortality data at the county-level from National Center for Health Statistics and population size from the U.S. Census Bureau.^17^ The life expectancy during 2002 to 2008 was estimated for males and females separately for each county. We used mean life expectancy between 2002 and 2008 for males and females for each county as the health outcome measure.

### Demographic Characteristics, Socioeconomic Status, and Health Risk Factors

The Tracking Network contains county-level data from the U.S. Census Bureau^18^ and the U.S. Department of Agriculture^19^ for linkage and analysis. We used county-level population, demographic, and socioeconomic data in 2002 which was considered as the baseline for assessing the temporal trends for ozone concentrations: total population, percentages of population aged 0 to 4 years and population aged 65 years or older, percentages of non-Hispanic whites, non-Hispanic blacks, and Hispanics, and percentage of adults with a high school or higher education. Population density (i.e., number of persons per square mile) in 2002 was calculated by dividing the population by the county area. The urban, suburban, and rural classifications were determined based on the Rural–Urban Continuum Codes Definitions for 2003 developed by the U.S. Department of Agriculture.^19^ County-level percentages of people of all ages in poverty (ie, a family's total income less than the poverty threshold) in 2002, and percentages of people aged 0 to 64 years without health insurance available for 2008 were estimated by the U.S. Census Bureau using a small area estimation method.^20,21^ The 2002 county-level unemployment rates were obtained from the U.S. Department of Labor.^22^ The prevalence of current smoking, obesity, no leisure-time physical activity, and self-rated poor or fair health in 2008 were estimated by a multilevel regression and poststratification method using data from the Behavioral Risk Factor Surveillance System.^23,24^ The 2008 model-based estimates were highly correlated with the BRFSS survey estimates for adult current smoking prevalence (*r* = 0.67; 2002–2008 BRFSS combined data among 2423 counties) and obesity prevalence (*r* = 0.70; 2006–2008 BRFSS combined data in 3109 counties), and prevalence of poor or fair health (*r* = 0.76; 2002–2008 BRFSS combined data in 2680 counties).

### Statistical Analysis

We first estimated the 7-year mean ozone concentrations from 2002 to 2008 for each county and examined their correlations with life expectancy using Spearman rank correlation coefficients (*r*_s_). We then conducted a latent class growth analysis (LCGA) to identify latent classes of counties based on the mean ozone concentrations and their rates of change overtime from 2002 to 2008 using the Mplus (version 7.0) program.^25^ The LCGA is a technique that examines the heterogeneity in mean levels and rates of change of longitudinal or repeated data. It is a useful tool to identify underlying clustering of counties with distinct patterns of ozone concentration.^26,27^ The number of latent classes was determined by comparing the model fit indices of LCGA models. The model with a smaller Bayesian Information Criterion value, large entropy (close to 1.0), and high posterior probability (close to 1.0) suggests a better fit to the data. A small *P* value (*P* *<* 0.05) for the Lo-Wendell-Rubin adjusted likelihood ratio test indicates that a model with one less class (k − 1) has to be rejected in favor of a model with at least k classes.^25–27^

We conducted further analyses to examine the differences in life expectancy and all other selected variables across different latent classes of ozone. First, we computed the percentages or means and tested equality across the 3 latent classes. The overall differences in means were tested using Wald F-tests. We used 2-sample *t-*tests with Bonferroni adjustment for *P* values to compare means between each paired groups and used nonparametric tests for testing the differences in medians. We conducted linear regression analyses to estimate the differences in life expectancy by ozone latent classes in 3 regression models: model without adjustments, model adjusted for PM_2.5_ concentration, and model adjusted for all selected covariates. We also conducted linear regression analyses using the overall mean ozone concentrations from 2002 to 2008 on mean life expectancy.

Based on previous research findings and theoretical considerations, we included the following variables as potential confounders in the multivariable analyses: PM_2.5_ concentrations (continuous scale, μg/m^3^), ozone monitoring seasons (3 categories: 1 = spring-summer season, 4 to 6 months, beginning in April or May or June, ending in September; 2 = spring-fall season, 7 to 9 months, beginning in March or April, ending in September or October or November; and 3 = full-year, 12 months, beginning in January, ending in December), annual mean days of heat index above 90°F (continuous scale, per day), urban and suburban classification (3 categories: 1 = urban, 2 = suburban, and 3 = rural), population density (continuous scale, per person per square mile), percentage of population age 0 to 4 years (continuous scale, %), percentage of population age ≥65 year (continuous scale, %), percentage of non-Hispanic blacks (continuous scale, %), percentage of Hispanics (continuous scale, %), percentage of people of all age in poverty (continuous scale, %), unemployment rate (continuous scale, %), percentage of people age 0 to 64 years without health insurance (continuous scale, %), prevalence of self-rated poor or fair health (continuous scale, %), current smoking (continuous scale, %), obesity (continuous scale, %), and no leisure-time physical activity (continuous scale, %) at the county level. Population density, percentage of non-Hispanic blacks, and percentage of Hispanics were log-transformed to approximate normal distribution.

The SAS System for Windows (Release 9.3) (SAS Institute Inc., Cary, NC) was used for descriptive and linear regression analyses. We considered results of 2-tailed *t*-tests to be statistically significant if *P* values were <0.05, and results of 2-tailed *t*-tests used in multiple comparisons to be statistically significant if *P* values were <0.01 (equivalent to *P* values <0.05 after Bonferroni correction). We performed the analyses for males and females separately in line with the sex differences in life expectancy.

## RESULTS

The overall annual mean daily 8-hr maximum ozone concentrations in 3109 counties decreased from 46.8 ppb (ranging from 22.5 to 72.7 ppb) in 2002 to 44.6 ppb (ranging from 29.3 to 64.5 ppb) in 2008 (*P* < 0.001). The 7-year mean seasonal ozone concentrations were significantly correlated with mean life expectancy in both males (*r*_s_ = −0.19; *P* < 0.0001) and females (*r*_s_ = −0.24; *P* < 0.0001). Ozone concentration moderately correlated with PM_2.5_ concentration (*r*_s_ = 0.50).

**FIGURE 1:**
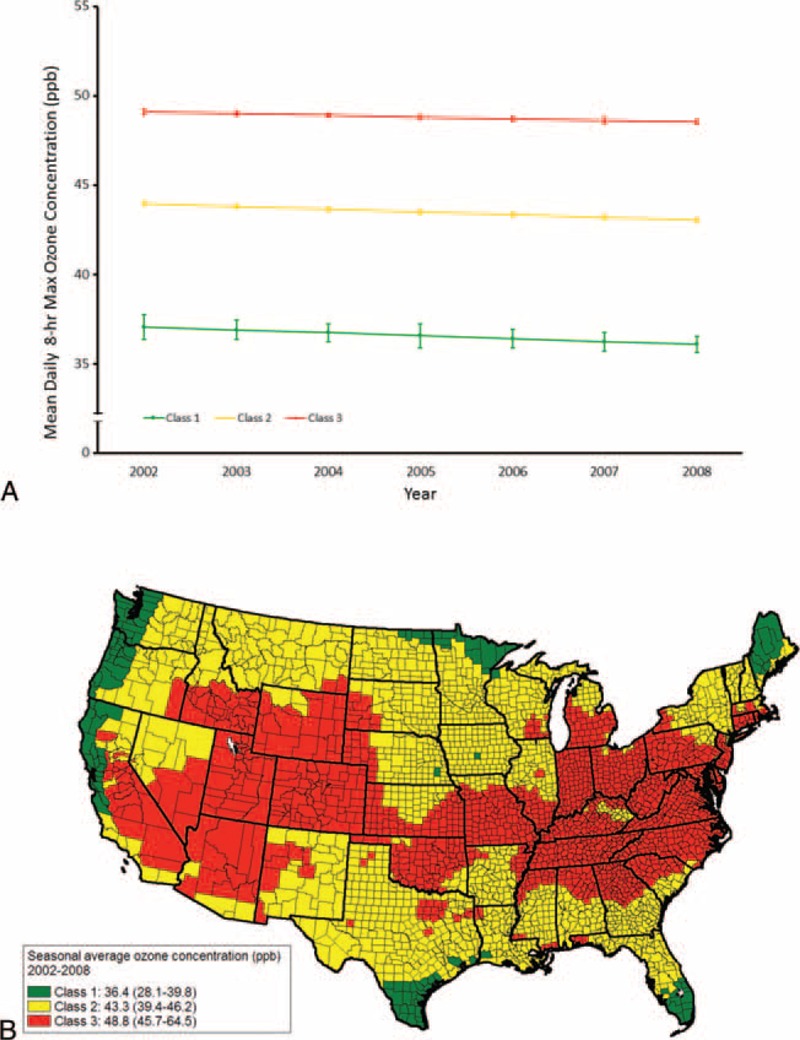
Mean ozone concentrations (ppb) and their 95% confidence intervals by latent classes (A) and geographic distribution of the class 1, class 2, and class 3 counties (B) in the conterminous United States.

The 3109 counties were classified into 3 classes with distinct mean levels and the rates of change for ozone concentrations during 2002 and 2008 (Figure 1A). Class 1 comprised 126 (4.1%) counties with a mean ozone concentration of 36.4 ppb (Figure 1B). Class 2 comprised 1450 (46.6%) counties with a mean of ozone concentration of 43.3 ppb. Class 3 comprised 1533 (49.3%) counties with a mean ozone concentration of 48.8 ppb. Statistically significant reduction in mean ozone concentrations did occur during 2002 and 2008, with a mean rate of reduction of −0.15 ppb (95% confidence interval [CI]: −0.17 to −0.13 ppb), −0.16 ppb (95% CI: −0.24 to −0.09 ppb), −0.15 ppb (95% CI: −0.18 to −0.13 ppb), and −0.09 ppb (95% CI: −0.12 to −0.07 ppb) in all counties, class 1 counties, class 2 counties, and class 3 counties, respectively (Table 1).

**TABLE 1:**
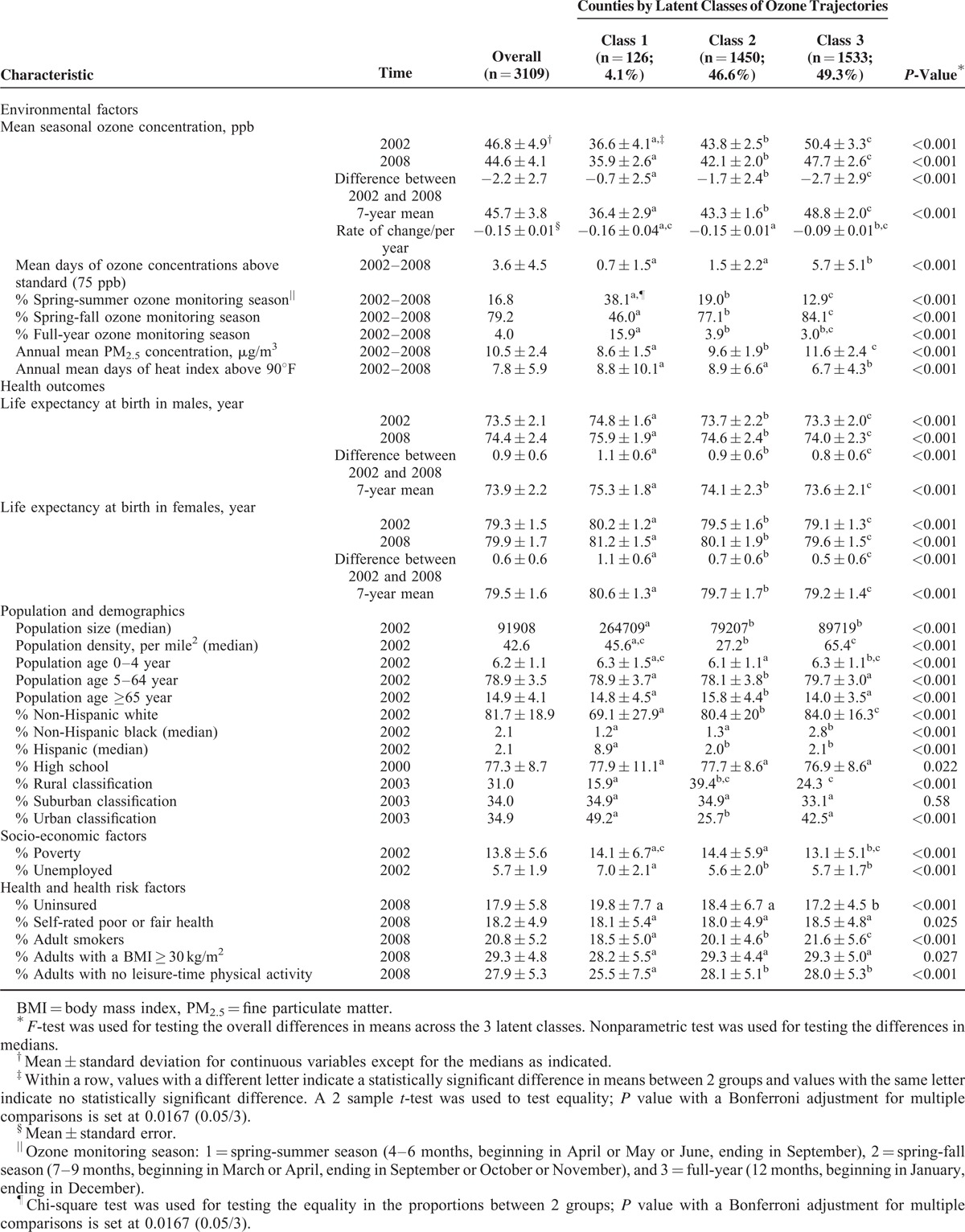
Characteristics of the 3109 Counties of the Conterminous United States by Ozone Latent Classes, 2002 to 2008

In 2002, the mean life expectancy was 73.5 years (ranging from 64.6 to 79.7 years) in males and 79.3 years (ranging from 73.7 to 83.4 years) in females. In 2008, the mean life expectancy was 74.4 years (ranging from 66.4 to 81.4 years) in males and 79.9 years (ranging from 74.0 to 86.0 years) in females. From 2002 to 2008, life expectancy increased 0.9 years (*P* < 0.0001) in males and 0.6 years (*P* < 0.0001) in females (Table 1). Overall, statistically significant differences existed in selected population and demographic characteristics, socioeconomic status, and health risk factors across the 3 classes of counties except percentages of suburban counties (Table 1).

In the regression model without adjustment for any covariates, life expectancy in the class 2 counties and the class 3 counties was 1.2 years (*P* < 0.0001) and 1.7 years (*P* < 0.0001) lower than in the class 1 counties in males, respectively, and was 0.9 year (*P* < 0.0001) and 1.4 years (*P* < 0.0001) lower in females, respectively (Table 2, model 1). After adjustment for PM_2.5_ concentration, the differences in life expectancy between the class 2, the class 3, and the class 1 counties decreased, but remained statistically significant in both males and females (all *P* < 0.0001) (Table 2, model 2). After further adjustment for all selected covariates, the differences in life expectancy between the class 2, the class 3, and the class 1 counties decreased to 0.2 and 0.6 year in males, and 0.3 and 0.6 year in females, respectively (all *P* < 0.0001) (Table 2, model 3).

**TABLE 2:**
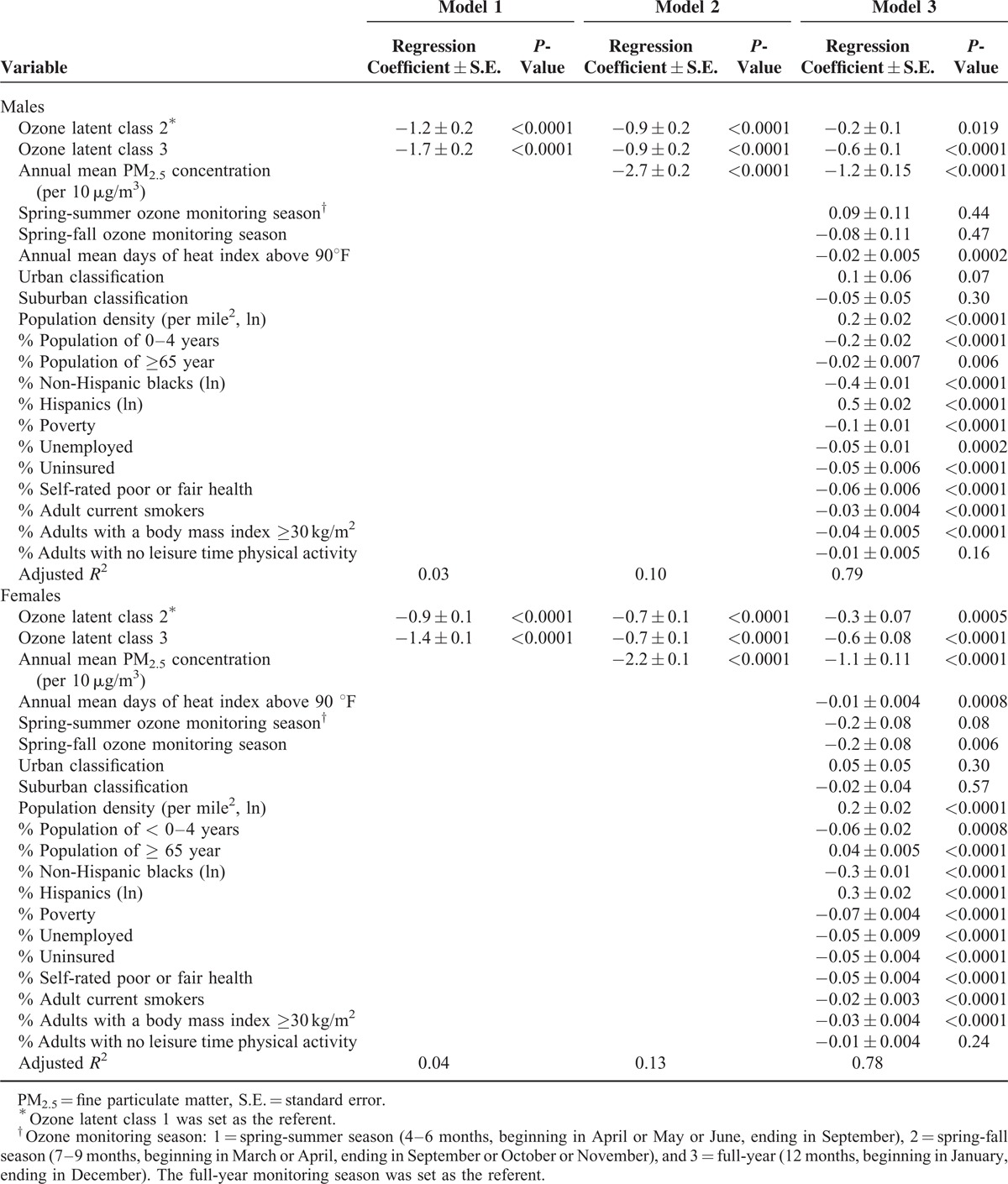
Estimated Differences in Life Expectancy by Ozone Latent Class and Selected Environmental, Demographic, Socioeconomic, and Health Indicators in Counties of the Conterminous United States (n = 3109), 2002 to 2008

**FIGURE 2:**
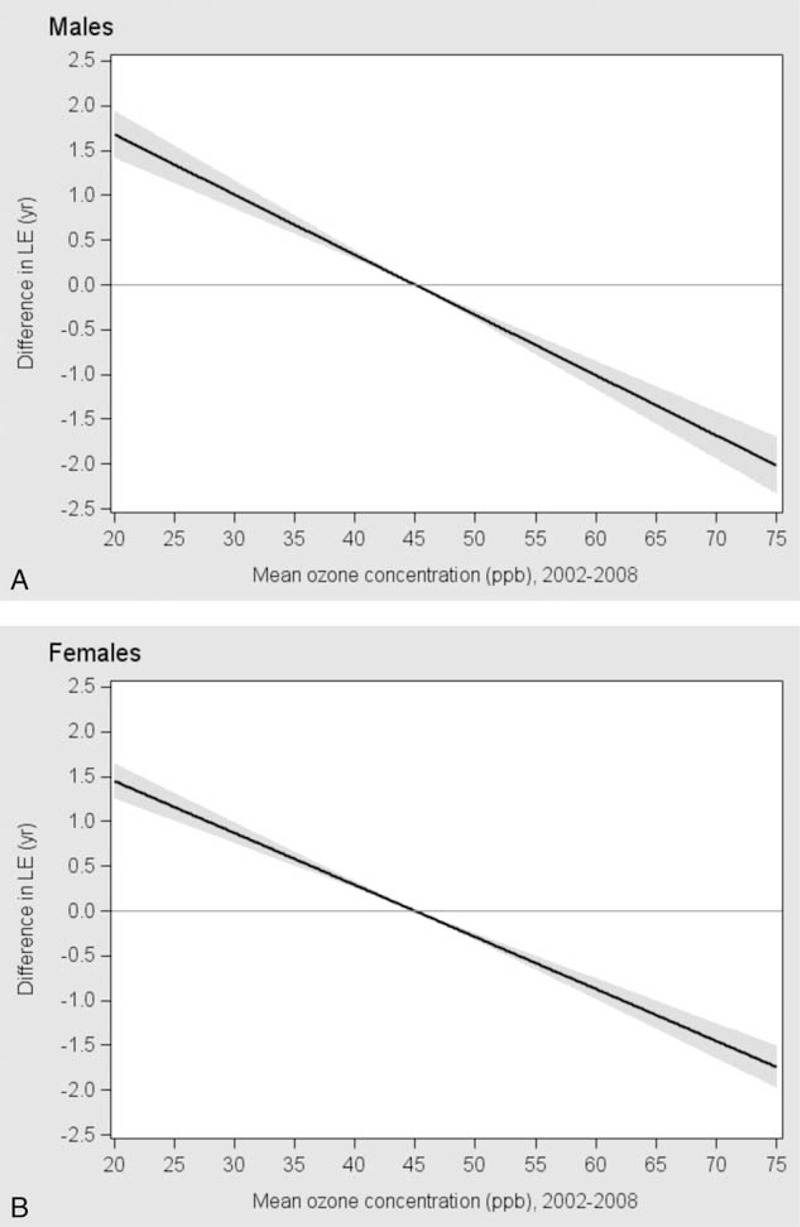
Exposure-response relationship between ozone concentrations (ppb) and differences in life expectancy in 3109 counties of the conterminous United States. The line is based on a linear regression analysis, adjusted for PM_2.5_ concentrations, days of heat index above 90°F, urban and suburban classification, percentage of population age 0 to 4 years, percentage of population age 65 year or older, percentage of non-Hispanic blacks, percentage of Hispanics, population density, percentage of uninsured, percentage of unemployed, prevalence of current smoking, prevalence of obesity, and prevalence of no leisure-time physical activity. Population density, percentage of non-Hispanic blacks, and percentage of Hispanics were log-transformed to approximate normal distribution. The solid line represents the estimates of difference in life expectancy between given ozone value compared to a reference ozone concentration of 45.0 ppb (the grand mean ozone concentration). The shaded area represents 95% confidence interval of the estimates.

The exposure-response curve shows that when compared with a reference level of ozone concentrations at 45.0 ppb (the national mean level), counties with an ozone concentrations above 45.0 ppb had a lower life expectancy and those with an ozone concentrations below 45.0 ppb had a higher life expectancy in both males (Figure 2A) and females (Figure 2B). Linear regression analysis results indicated that after controlling for potential confounding effects of demographic and socio-economic characteristics and health risk factors, a 5 ppb higher ozone concentration was associated with 0.25 year lower life expectancy in males (95% CI: −0.30 to −0.19) and 0.21 year in females (95% CI: −0.25 to −0.17).

To account for the possible unstable estimates for life expectancy in counties with small population, sensitivity analyses were conducted by repeating the multivariable regression models in the subsample of counties with a population size <15,000 (n = 1024), 15,000 to <45,000 (n = 1080), and ≥45,000 (n = 1005). The associations between ozone concentrations and life expectancy were similar across the analyses of subsamples (Table 3).

**TABLE 3:**
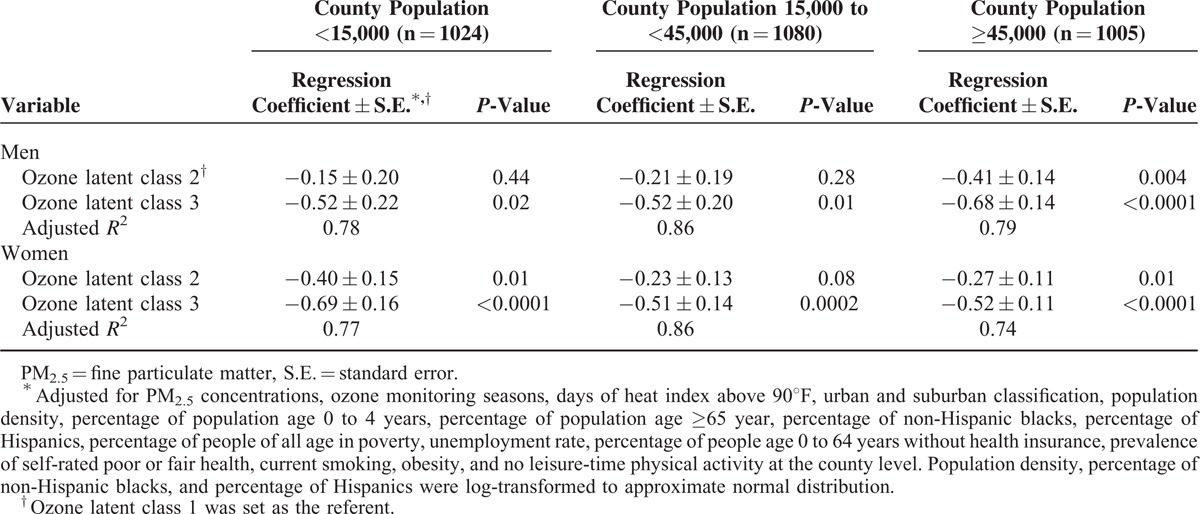
Estimated Differences in Life Expectancy by Ozone Latent Class Stratified by County Population Size and Sex, 2002 to 2008

## DISCUSSION

In this ecological study of all 3109 counties of the conterminous U.S., we found an overall decreasing trend in mean ozone concentrations over the 7-year period, albeit the rate of change was small (−0.15 ppb per year). We identified 3 classes of counties with distinct developmental trajectories of ozone concentrations between 2002 and 2008. The class of counties with the persistently highest mean ozone concentrations had about 1.7- and 1.4-year lower life expectancy in males and females, respectively, when compared with the class of counties with the lowest ozone concentrations.

To the best of our knowledge, this is the first study that assessed the direct association between long-term exposure to ozone and life expectancy using data from all counties in the conterminous U.S. In addition, we used a state-of-the-art statistical model to capture the county-level heterogeneities in the mean ozone concentrations and the rate of change over a period of 7 years. Although the LCGA has been primarily applied to psychosocial and behavioral studies.^26–28^ its application in environmental health has been scarce. Our analyses demonstrated that this method was useful for identifying the latent classes of counties that had persistently high ozone concentrations overtime, a practical approach for identifying long-term exposure to ozone overtime. The significant association between ozone concentrations and life expectancy found in this study add support on the potential adverse effects of elevated ozone concentrations on human health.

Because life expectancy at birth is a composite measure of population health, increase in mortality among infants, children, and adults could contribute to decrease in life expectancy. A previous study showed that exposure to ozone is associated with increased risk of infant mortality,^14,29^ and mortality from respiratory diseases and cardiovascular diseases in adults.^6,30–34^ In addition, exposure to ozone has been associated with increased hospitalization and admission to emergency department due to asthma and chronic obstructive pulmonary disease,^2–5^ which could potentially increase the risk of mortality. Although the exact mechanisms linking long-term exposure to ozone and life expectancy remains unclear, laboratory and clinical studies have shown that acute exposure to ozone is linked to worsening respiratory symptoms, reducing pulmonary functions, and inflammatory responses.^35,36^

Pope et al^9^ analyses of 211 counties in 51 U.S. metropolitan areas during 1980 to 2000 found that a decrease of 10 μg/m^3^ in PM_2.5_ concentration was associated with an increase in 0.6-year life expectancy. Our results also showed that every 10 μg/m^3^ increase in PM_2.5_ concentration was associated with about 1.2- and 1.0-year decrease in life expectancy in males and females, respectively, independent of ozone and other selected demographic characteristics, socioeconomic status, and health risk factors. Our data showed that PM_2.5_ moderately correlated with ozone. Therefore, it is possible that PM_2.5_ may partially confound the association between ozone and life expectancy.

Our results that life expectancy was lower in the counties with a higher ozone concentration than that in the counties with a lower ozone concentration add support for the possible adverse health impact of elevated ground-level ozone concentrations. By using a health impact assessment model, a recent study demonstrated that by achieving the National Ambient Air Quality Standard (NAAQS) for ozone, substantial numbers of ozone-related premature deaths could be avoid in the U.S.^37^ In 2008, the U.S. EPA lowered the primary NAAQS for daily 8-hr max ozone concentration to 75 ppb and has proposed to revise the standard to a level within the range of 60 to 70 ppb.^38^ Indeed, with the lower NAAQS standards for ozone, greater health benefits would have resulted.^37^ Another previous study indicated that even low ground-level ozone concentrations are associated with increased risk of premature death.^39^

Our results are subject to some limitations. First, we based our analyses on ecological data; we therefore were unable to make causal inferences. We selected known covariates to adjust for their potential confounding effects on the association between ozone and life expectancy. Because about 80% of variations in life expectancy were accounted for by ozone and selected covariates together, residual confounding of unmeasured environmental, socio-economical, and health-related factors could be minimal. Since our results were based on aggregated data at county level, cautions may be needed when one makes an inference to subcounty level due to possible ecological fallacy. Second, we were unable to account for possible geographic mobility of the population in the 7-year period. Third, for ozone concentrations in counties without monitors, we used model-based data rather than actual measured data in our analyses, which could potentially introduce bias in the results. However, such bias could be minimal: in counties with the monitors, the model-based data have been highly correlated with the monitor-based data (*r* ranges from 0.61 to 0.86).^40^

In summary, by using data from all 3109 counties of the conterminous U.S., we identified 3 classes of counties with distinct mean levels and rates of change in ozone concentrations between 2002 and 2008. Our findings suggest that independent of PM_2.5_, temperature, and other known demographic, socio-economic, and health risk factors, long-term exposure to a higher ozone concentration may be associated with a lower life expectancy.
